# MAGI1 localizes to mature focal adhesion and modulates endothelial cell adhesion, migration and angiogenesis

**DOI:** 10.1080/19336918.2021.1911472

**Published:** 2021-05-06

**Authors:** Begoña Alday-Parejo, Kedar Ghimire, Oriana Coquoz, Gioele W. Albisetti, Luca Tamò, Jelena Zaric, Jimmy Stalin, Curzio Rüegg

**Affiliations:** a Laboratory of Experimental and Translational Oncology, Pathology, Department of Oncology, Microbiology and Immunology, Faculty of Sciences and Medicine, University of Fribourg, Fribourg, Switzerland; b Westmead Institute for Medical Research, University of Sydney, Sydney, Australia; c Institute of Pharmacology and Toxicology, Section of Neuropharmacology, University of Zürich, Zürich, Switzerland; d Clinical Trials Unit, University of Bern, Bern, Switzerland; e Swiss Institute for Experimental Cancer Research, Ecole Polytechnique Fédérale De Lausanne, Lausanne, Switzerland

**Keywords:** Cell adhesion, migration, signaling, extracellular matrix, cytoskeleton, stress fibers

## Abstract

MAGI1 is an intracellular adaptor protein that stabilizes cell junctions and regulates epithelial and endothelial integrity. Here, we report that that in endothelial cells MAGI1 colocalizes with paxillin, β3-integrin, talin 1, tensin 3 and α-4-actinin at mature focal adhesions and actin stress fibers, and regulates their dynamics. Downregulation of MAGI1 reduces focal adhesion formation and maturation, cell spreading, actin stress fiber formation and RhoA/Rac1 activation. MAGI1 silencing increases phosphorylation of paxillin at Y118, an indicator of focal adhesion turnover. MAGI1 promotes integrin-dependent endothelial cells adhesion to ECM, reduces invasion and tubulogenesis*in vitro* and suppresses angiogenesis  *in vivo*. Our results identify MAGI1 as anovel component of focal adhesions, and regulator of focal adhesion dynamics, cell adhesion, invasion and angiogenesis.

## Introduction

Endothelial cells (EC) maintain the vascular barrier function by establishing a set of intercellular adhesion, namely tight junctions, adherens junctions and gap junctions [1], but also by establishing adhesive interactions with the surrounding extracellular matrix (ECM) [2]. Cell adhesion to the ECM is mediated by a class of cell surface receptors called integrins [3]. Integrins are heterodimeric transmembrane receptors composed by an α- and a β-chain that are activated from a low affinity to a high affinity ligand-binding state upon the recruitment of the cytoplasmic adaptor proteins talin and kindlin to the β-integrin cytoplasmic tail (‘inside-out signaling’) [4,5]. High-affinity ligand binding will induce, in turn, further conformational changes of the integrin cytoplasmic tails promoting the recruitment of a series of integrin-associated proteins (the adhesome) that tether the cytoskeleton to form nascent adhesion complexes, also called focal complexes [6,7]. Proteins recruited to focal adhesion complexes include actin-associated proteins such as vinculin or α-4-actinin, and scaffolding and signaling proteins, including paxillin and focal adhesion kinase (FAK) [7,8]. Focal complexes can then, either disassemble to allow focal adhesion turnover and cell migration, or mature in an actomyosin–dependent manner into large and stable focal adhesions clustering at the cell surface, where they interact with additional cytoplasmic linker proteins to further stabilize cell adhesion [8–11]. In turn, integrins at focal adhesions transmit biochemical signals from the ECM to the cell that regulate essential cellular processes such migration, proliferation, differentiation and survival (‘outside-in signaling’) [12,13].

MAGI1 (Membrane associated guanylate kinase, WW and PDZ domain containing 1) is a member of the large family of MAGUK (Membrane-associated guanylate kinases) proteins consisting of six PSD95/DiscLarge/ZO–1 (PDZ) domains, a guanylate kinase domain and two WW domains. As in other MAGUK proteins, MAGI1 guanylate kinase domain is catalytically inactive [14]. MAGI1 is recruited to tight junctions and adherens junctions thereby connecting them with the actin cytoskeleton though a scaffolding function [15–19]. For example, MAGI1 colocalizes with ZO-1 and α-4-actinin at tight junctions at the apical part of epithelial cells [17,18] and with E-cadherin, β–catenin and PTEN at the basolateral adherens junctions [20–22].

In ECs, MAGI1 was reported to stabilize intercellular adhesion [23] and to regulate eNOS expression and vascular NO production in response to fluid shear stress [24], processes essential to vascular function. However, little is known about MAGI1 interaction with other adhesive structures like focal adhesions and the regulation of endothelial cells’ adhesion and migration. Here we report that in endothelial cells, MAGI1 colocalizes at focal adhesions and regulates their dynamics by stabilizing their maturation while preventing turnover. These effects translate into increased endothelial cell adhesion to the ECM, and reduced endothelial cell migration, tube formation and angiogenesis.

## Materials and methods

### Antibodies

#### Primary antibodies

Anti-MAGI1 mouse monoclonal clone 7B4 and 3B4, Anti-MAGI1 rabbit polyclonal M5691 were from Sigma-Aldrich. Immunostainings were performed with mAb7B4 and 3B4 while western blots were performed using the anti-MAGI1 rabbit polyclonal antibody. Anti-β3-integrin rabbit ab75872 (Abcam); Anti-phospho-paxillin Y118 rabbit (Cell signaling); Anti-talin 1 rabbit ab71333 (Abcam), Anti-β1-integrin rabbit; Anti-α-4-actinin rabbit ab108198 (Abcam); Anti-paxillin mouse clone 349 (BD Biosciences); Anti-PTP-PEST (D4W7W) rabbit (Cell signaling); Anti-GAPDH rabbit G9545 (Sigma-Aldrich). *Directly labeled antibodies*: Anti-α-4-actinin rabbit-Alexa Fluor 647 ab198610 (Abcam); Anti-tensin 3 PA5-63,112; Phalloidin-Alexa Fluor 568 (Thermo Fisher Scientific).

#### Secondary antibodies

HRP-conjugate, Goat anti-rabbit and Goat anti-mouse (DAKO). Donkey anti-mouse Alexa Fluor 488 A-21,202 (for MAGI1) (Thermo Fisher Scientific), Goat anti-rabbit Alexa Fluor 546 A-11,071 (for β3-integrin, β1-integrin, phospho-paxillin, talin 1, α-4-actinin) (Thermo Fisher Scientific), Donkey anti-rabbit-Alexa Fluor 405 ab175651 (for phospho-paxillin) (Abcam).

### Cell culture

Primary Human umbilical vein endothelial cells (HUVECs) were isolated from human umbilical cords as previously described [25]. HUVEC were cultured in M199 supplemented with Glutamax, 10% FCS, 12 µg/ml of bovine brain extract (Clonetics), 10 ng/ml recombinant epidermal growth factor (EGF) (Genzyme), 25 U/ml heparin, 1 µg/ml hydrocortisone (Sigma) and 1% penicillin/streptomycin. Ethic committee for human experimentation of Canton Vaud (Switzerland) approved collection and use of HUVEC (CER-VD_105/04). Human embryonic kidney cells expressing a mutant version of the SV40 large T antigen (HEK293T) were obtained from American Type Culture Collection (ATCC) and were cultured in Dulbecco’s modified Eagle’s medium (DMEM) supplemented with 10% FCS and 1% penicillin/streptomycin. All cells were maintained in a humidified incubator at 37°C and 5%CO2.

### Immunofluorescence staining and confocal microscopy

HUVECs were seeded on gelatin-coated glass coverslips in a 12-well plate and cultured to sub-confluency. Upon stimulation with 1 mM MnCl2, cells were washed with PBS, fixed in 4% PFA for 15 min at 4°C, blocked and permeabilized (0.5% BSA, 5% donkey serum, 0.1% TritonX-100 in PBS) for 30 min. Cells were incubated with primary antibodies for 1 hour at room temperature, washed with PBS 4–5 times and incubated with relevant fluorescent-conjugated secondary antibodies. Cell nuclei were counterstained and mounted in ProLongTM Gold antifade reagent with DAPI (4,6-diamidino-2-phenylindole) (Thermo Fisher Scientific). Images were acquired with a Leica TCS SP5 inverted confocal laser scanning microscope (Leica Microsystems) using a 63x objective and with a pinhole of 1 AU to minimize z-section.

### Quantification of focal adhesions and cell spread area

Immunofluorescence staining from cultured cells was acquired by confocal microscopy and analyzed by Fiji (Image J). Focal adhesion quantification was performed on paxillin immunostainings as previously described [26]. Based on their work we stablished a cutoff of 50 pixels as a threshold to separate focal complexes/adhesions from background noise. The cutoff to define focal complexes *vs* growing focal adhesions *vs* mature focal adhesions was based on the work of Kim and Wirtz [27] Minor modifications of the protocol were made to adjust it to cell lines with different sizes, e.g. 4.16 pixels/µm scale for HUVEC and 8.32 pixels/µm scale for 293 T cells. When subtracting the background in sliding paraboloid option, a rolling ball radius of 50 was set in HUVEC and of 25 in 293 T cells. Definition of paxillin-positive focal structures by size was the following [27]: nascent focal complexes (FXs): <1 µm^2^; growing focal adhesions (gFA): 1– 3 µm^2^; mature focal adhesions (mFA): >3 µm^2^. Size distribution analysis in control HUVEC revealed that 64.8% of the structures were below 1 µm^2^ (FXs), 31.7% between 1 and 3 µm^2^ (nFAs) and 3.4% above 3 µm^2^ (mFAs) in size.

For cell spreading and morphology measurements, we analyzed paxillin immunostaining by Fiji. First, we adjusted the contrast threshold until the borders of the cells were well defined and then we applied the oval selection tool to individual cells to measure circularity and area of circularity. For all quantifications, 8 random and representative areas from the cell culture slides were analyzed per condition with a 63x magnification lens.

### Adhesion assay

NUNC Maxisorp II (NUNC) ELISA 96-well plates were coated with fibronectin (3 µg/ml), Collagen I (10 µg/ml), gelatin (0.5%) and laminin (derived from basal membranes of EHS sarcoma) (3 µg/ml) overnight at 4°C and blocked with 1% BSA for 2 hours at 37°C. 1% BSA-coated wells were used as negative controls and Poly-L-lysine- (PLL) coated wells were used as the positive control. HUVEC were collected by trypsin digestion and seeded in serum-free M199 medium at 2 × 10^4^ cells/well in 100 µl volume. The plate was incubated for 2 hours at 37°C. After incubation, non-attached cells were removed by gently washing with PBS. Attached cells were fixed with 4% PFA and stained with 0.5% crystal violet (CV) solution (3.75 g Crystal violet, 1.75 g NaCl, 61.5 ml EtOH in 500 ml water) for 1 hour. CV solution was gently washed with deionized water until negative control wells were clean. Once the plate was dried, CV was eluted with 100 µl/well of distaining solution (0.1% acetic acid/50% ethanol). Absorbance of each well was read at 620 nm in a plate reader (Tecan Infinite M200Pro). Results were normalized by BSA coated wells and expressed as mean values of triplicate measurements SD.

### RhoA and Rac1 activity assays

RhoA and Rac1 activities were assayed using an ELISA-based active GTPase capture assay (Cytoskeleton, Inc., Ref: BK124 and BK12) and following the recommended protocol. Briefly, HUVEC silenced for MAGI1 expression (sh1 and sh2) or overexpressing MAGI1, and their relative controls (non-silencing sh and empty vector-transduced HUVECs), were freshly plated on gelatin (0.5%) and fibronectin (3 µg/ml)-coated non-tissue culture plates. Assay was performed in triplicate conditions. At 1.5 or 24 hours after plating adherent HUVEC rinsed with ice-cold PBS, and lysed/scraped on ice in the lysis buffer provided with the kit. Lysates were cleared by centrifugation (10ʹ000xg, 4°C, 1 min), and supernatants were snap-frozen in liquid nitrogen and stored at −80 °C until use. After protein quantification, 12.5 µg of total protein for each sample was tested in duplicate for each well and conditions following the manufacturer’s protocols. Results are expressed as mean values ± S.D.

### Co-immunoprecipitation

293 T cells or HUVEC were lysed in cooled NP-40 lysis buffer (Sino biological) containing protease inhibitor cocktail (PI) and phosphatase inhibitors: phenylmethylsulfonylfluoride (PMSF), sodium orthovanadate (Na3VO4) and β-Glycerophosphate disodium salt hydrate (BGP). Protein lysates were passed through syringes and centrifuged at 13,000 rpm for 10 min at 4°C to remove cellular debris. Protein lysates were incubated overnight at 4°C and rotation with the corresponding primary antibodies e.g.: paxillin, PTP-PEST, β3-integin, talin 1, MAGI1 and the isotype control IgG antibody (5 µg per 200 µl cell lysate). Afterward, protein lysates were incubated with magnetic beads-protein G (Sino biological) for 3 hours at 4°C and rotation (40 µl of magnetic beads per 200 µl cell lysate). Before incubation with primary antibody and cell lysates, magnetic beads-protein G were pre-washed three times with 1x PBST. After incubation with primary antibody and cell lysates, magnetic beads bound to the protein complex of interest were washed three times with 1x PBS (containing PI) and supernatants 1 (S1), 2 (S2) and 3 (S3) were kept. Magnetics beads were eluted in 20 µl of elution buffer (Sino biological) and analyzed by western blot.

### In vitro invasion assay

Matrigel (0.5 mg/ml) was polymerized at 37°C in the upper Transwell chambers (8.0 µm pore size, Corning life sciences). Serum-starved (24 hours) HUVECs (4x10^4^) were seeded in the upper chamber and incubated in full medium for 24 hours. Filters were fixed with 4% PFA, stained with 0.5% CV and migrated cells counted in 3 random fields/membrane under a microscope. (n = 4) Values represent means ± S.D.

### In vitro 2D tubulogenic assay

A film of Matrigel (0.5 mg/ml) was polymerized at 37°C overnight in the bottom of 6 wells plates. HUVECs (5x10^4^) were seeded in the wells in complete medium and incubated for 4 hours. Cells were fixed with 4% PFA for 15 min at 4°C and closed pseudo-capillaries formed were counted under a microscope [28]. (n = 3). Values represent means ± S.D reported as percentage of control conditions (non-silenced or empty vectors transduced HUVEC)

### In vivo Matrigel plug assay

The assay was performed as previously described [29]. Briefly, 400 µl of growth-factor depleted Matrigel (Becton Dickinson) supplemented with PBS, 600 ng/ml FGF2 (Peprotech,), was injected into the flank of VEC_tA2:: tet_MAGI1 mice (DT) and control mice (FVB/N, and single transgenic mice, ST). Generation of mice was previously reported [24]. After 14 days mice were sacrificed and plugs evaluated for the presence of angiogenic vessels both macroscopically and biochemically by the Drabkin assay (hemoglobin content) [30]. The experiment was repeated three times, n = 3 mice.

### Statistical analysis

Normal distribution of samples was assessed by Shapiro–Wilk test. Data from *in vitro, ex vivo* and *in vivo* experiments were analyzed by Student’s t test or Mann–Whitney test when applicable. Results were considered significant with at least P < 0.05 (*), P < 0.01 (*), P < 0.005 (***), P < 0.001 (****). Results are expressed as mean ± SD unless otherwise indicated.

## Results

### MAGI1 colocalizes with phospho-paxillin, β3-integrin and talin 1 at large and mature focal adhesions in endothelial cells

**Figure 1.**
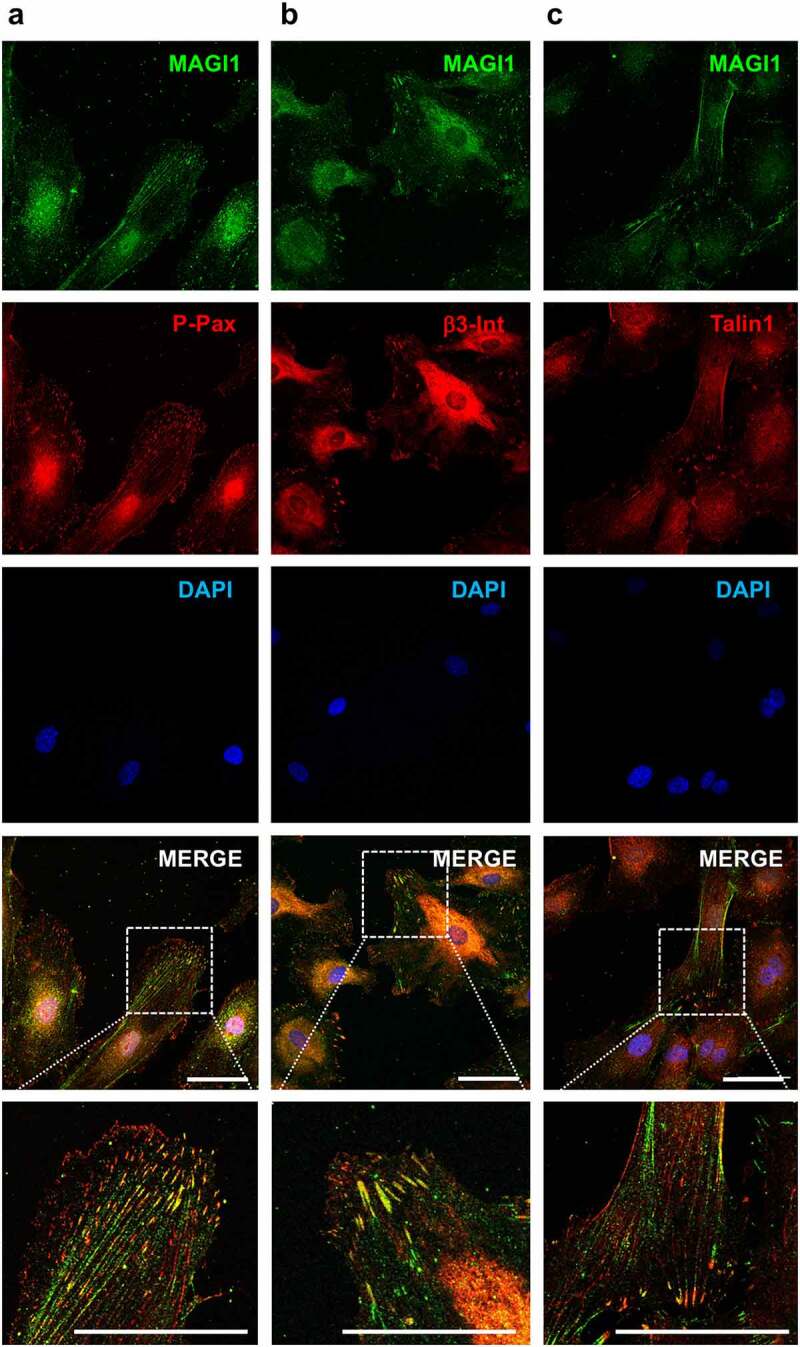
MAGI1 is present at large focal adhesions in endothelial cells. MAGI1 colocalizes with (a) phospho-paxillin, (b) β3-integrin and (c) talin 1 at large and mature focal adhesions in HUVEC. Confocal images of immunofluorescence staining of MAGI1 (green), phospho-paxillin, β3-integrin and talin 1 (red), DAPI (blue), and merged images in HUVEC (63x objective). Higher magnifications of selected regions of interest in the merged images are shown at the bottom. The scale bars correspond to 50 µm

In epithelial and endothelial cells, MAGI1 is known to localize at cell-cell adhesion membrane structures such as tight and adherens junctions, where it connects them to the actin cytoskeleton to stabilize intercellular adhesion **[**15,17,18,20,22**]**. However, it is not clear whether there is presence of MAGI1 at cell-ECM adhesion structures, such as focal adhesions. To address this question, we performed immunostainings in HUVEC and found that MAGI1 colocalizes with phospho-paxillin, β3-integrin and talin 1, three proteins typically present at focal adhesions (Figure 1a, 1b, 1c). Interestingly, MAGI1 only localized at large and mature focal adhesions but not at the small, dot-like, dynamic peripheral focal complexes, nor at centripetally located fibrillar adhesions (Figure S1). Fibrillar adhesions contain α5β1 integrins that are translocated centripetally out of peripheral focal adhesions **[**31,32**]**.

We conclude that MAGI1 protein is recruited to large focal adhesions, but not to small focal complexes or fibrillar adhesions.

### MAGI1 colocalizes with the actin associated protein α-4-actinin at actin stress fibers and focal adhesions in endothelial cells

**Figure 2.**
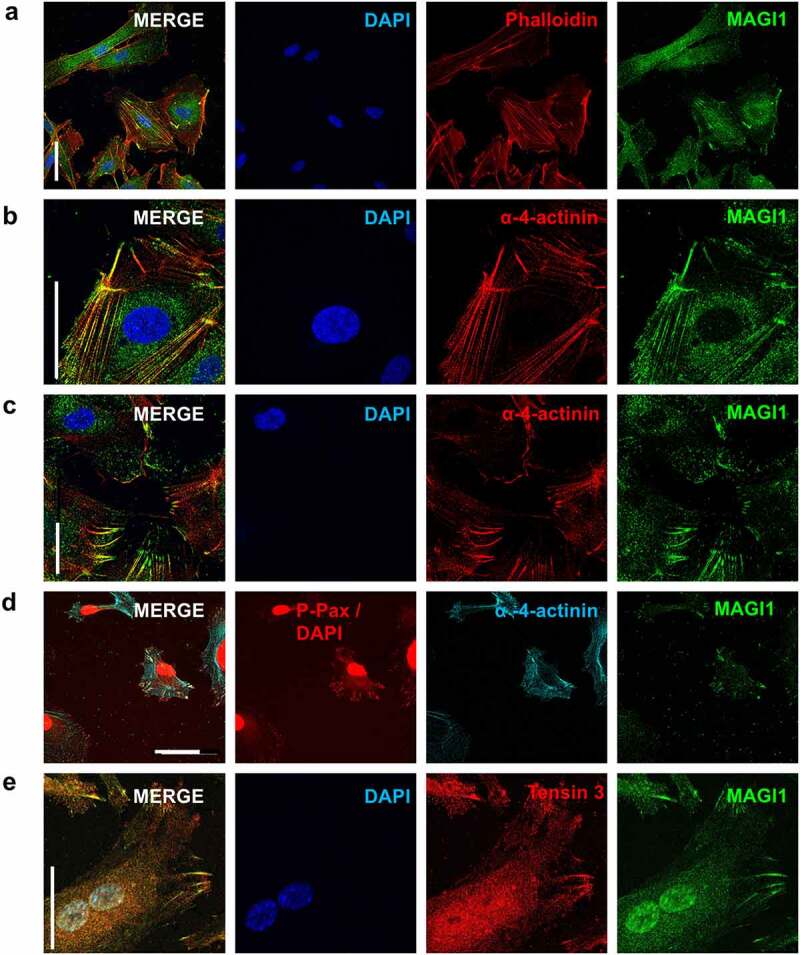
MAGI1 colocalizes with α-4-actinin at stress fibers and focal adhesions in HUVEC. (a-c). MAGI1 colocalizes along (a) phalloidin-positive actin stress fibers, (b) actin associated protein α-4-actinin along actin stress fibers, and (c) at the contacts between actin stress fibers and focal adhesions. Confocal images of immunofluorescence staining of MAGI1 (green), phalloidin and α-4-actinin (red), DAPI (blue), and merged images in HUVEC (63x objective, 1.5x zoom for α-4-actinin staining). (d). Confocal images of immunofluorescence staining of MAGI1 (green), phospho-paxillin and DAPI (red) and α-4-actinin (cyan), and merged images in HUVEC. Nuclear DAPI fluorescence was acquired with the same channel as for phospho-paxillin and arbitrarily set as red. (e). Confocal images of immunofluorescence staining of MAGI1 (green), tensin 3 (red), and merged images in HUVEC (63x objective, 1.25x zoom. Scale bars correspond to 50 µm

Further, we observed that MAGI1 staining also appeared as a fiber-like staining resembling actin stress fibers starting at focal adhesions and spanning through the cell **[**33**]** (Figure 1a and 1c). To study this observation in more detail, we performed double HUVEC immunostainings of MAGI1 and polymerized actin (phalloidin positive) and found that MAGI1 colocalizes with actin stress fibers but not with cortical actin (Figure 2a). MAGI1 also colocalizes with the actin-associated protein α-4-actinin along actin stress fibers (Figure 2b) and at the contact points between actin stress fibers and focal adhesions (Figure 2c). Triple immunostaining of MAGI1, phospho-paxillin and α-4-actinin, confirmed the colocalization of MAGI1, phospho-paxillin and α-4 actinin at focal adhesions (Figure 2d). MAGI1 also co-localized with tensin 3 (Figure 2e), an adaptor protein present in mature focal adhesion **[**34**]**, providing further evident for its recruitment to mature, β3-integrin containing focal adhesions.

To collect biochemical evidence for the presence of MAGI1 at focal adhesions, we performed co-immunoprecipitation experiments on lysates of adherent HUVECs by immunoprecipitating MAGI1, talin 1 and anti-β3-integrin and revealing the presence of talin 1 or MAGI1 in the captured material by western blotting. These experiments revealed that anti-MAGI1 antibody co-precipitated talin 1 and that anti-β3-integrin antibody co-precipitated MAGI1, compared to IgG control antibodies, consistent with the presence of MAGI1 in β3-integrin- and talin-1-containing complexes (Figure S2).

Taken together these results demonstrate that MAGI1 co-localizes with the actin-associated protein α-4-actinin along actin stress fibers, and with phospho-paxillin, α-4-actinin and tensin 3 in mature focal adhesions.

### MAGI1 downregulation reduces focal adhesion formation, cell spreading and actin stress fiber formation

**Figure 3.**
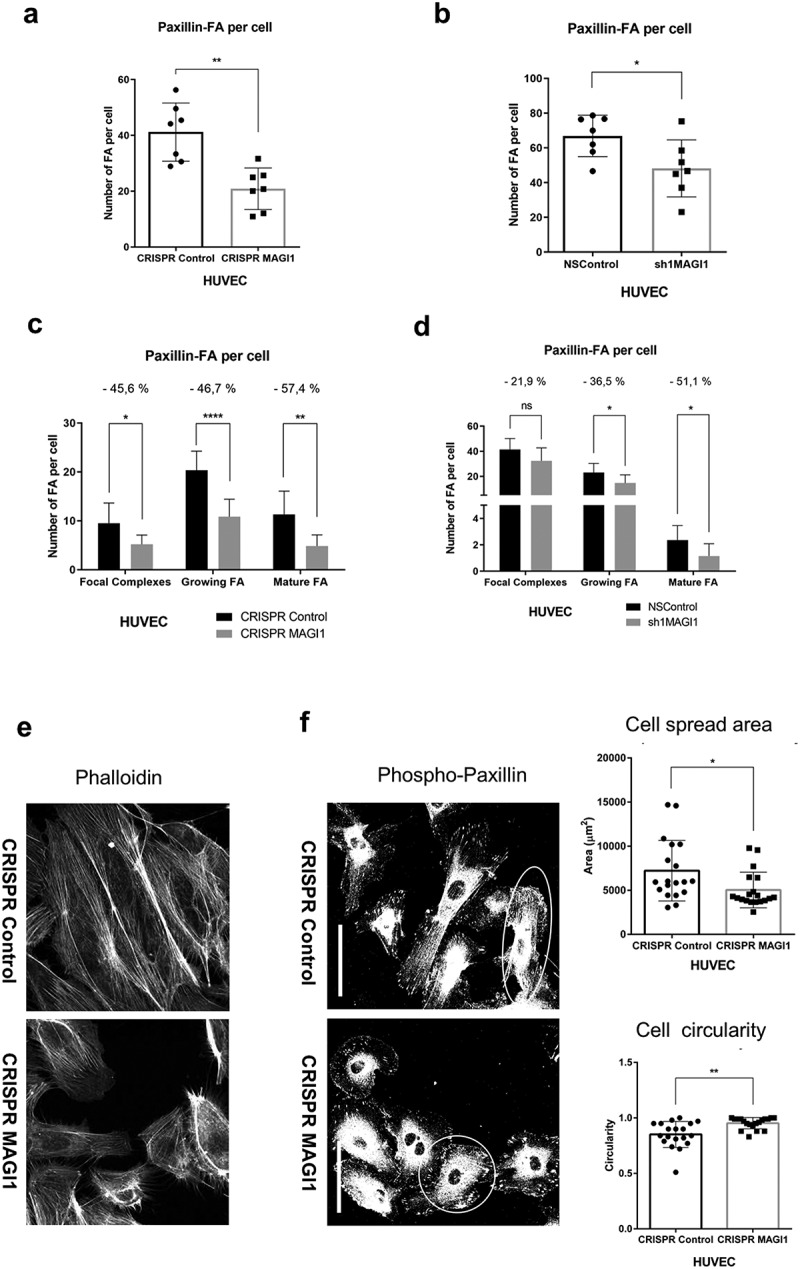
MAGI1 downregulation in HUVEC reduces focal adhesion formation, cell spread and formation of actin stress fibers. (a-b). Quantification of total number of paxillin-positive focal adhesions (FA) per cell in HUVEC with downregulated MAGI1 by (a) CRISPR/Cas9 or (b) shRNA and their relative controls. (c-d). Quantification of number of paxillin-stained focal adhesions (FA) per cell by group size in HUVEC with downregulated MAGI1 by (c) CRISPR/cas9 or (d) shRNA and their relative controls. Nascent adhesions or focal complexes (FXs) (<1 µm2), growing focal adhesions (FAs) (1– 3 µm2) and mature FAs (>3 µm2). The data represents mean values ± S.D. (n = 7). (e). Confocal immunofluorescence images of phalloidin (red) and DAPI (blue) in HUVEC control or with down-regulated MAGI1 by CRISPR/Cas9 (63x objective). (f). Representative images and quantification of cell spread area and circularity in paxillin-positive HUVEC control or HUVEC with down-regulated MAGI1 by CRISPR/Cas9 (63x objective). Scale bars correspond to 50 µm. The data represents mean values ± S.D. (n = 19). Statistical analyses were performed by unpaired t-test. ns = no statistical difference, * P < 0.05, *** P < 0.005, **** P < 0.001

In order to test whether MAGI1 has any functional effects on focal adhesions, we first downregulated its expression in HUVEC by shRNA and CRISPR/Cas9 (Figure S3a, S3b). Consistently, we observed that MAGI1 downregulation led to a significant reduction in the total number of paxillin-positive focal adhesions (Figure 3a and 3b). Since immunostaining colocalizations revealed that MAGI1 is rather located at large and mature focal adhesions (Figures 1 and 2), in order to obtain information on whether MAGI1 affects focal adhesion maturation, we classified adhesions complexes based on size, as following: nascent adhesions or focal complexes (FXs): <1 µm^2;^ growing focal adhesions (FAs): 1– 3 µm^2^; mature FAs:>3 µm^2^ **[**27**]** (see Materials and Methods section form more details). This analysis revealed that downregulation of MAGI1 reduces the number of all focal complexes and focal adhesions, but especially of the growing and mature focal adhesions (Figure 3c and 3d). To test whether high MAGI1 expression would have the opposite effect, we overexpressed MAGI1 in HUVEC by cDNA transduction (Figure S3a, S3b). MAGI1 overexpression lead to an increase in the number of paxillin-positive focal adhesions, both in total and by size groups (Figure S3c), however, the increase was not statistically significant.

As focal adhesions mature, they change their function from dynamic transmitters of strong propulsive forces necessary for migration to more static anchorage devices for maintaining cells attached and spread on the substrate **[**35**]**. Based on these considerations, we analyzed whether MAGI1 modulation in HUVEC also affected actin-stress fiber formation and cell morphology. To this end, we stained HUVEC with paxillin and phalloidin and monitored their shape by calculating cell circularity and cell spread area. Indeed, MAGI1 downregulation lead to a decrease of actin stress fibers compared to control cells displaying a well develop actin stress fibers (Figure 3e). MAGI1 downregulation rendered cells more circular and less spread compared to control cells (Figure 3f). Conversely, HUVEC with MAGI1 overexpression were larger, more spread but retained circularity compared to pLentiMOCK control HUVEC (Figure S3d). Importantly, we previously reported that MAGI1 silencing (or overexpression) does not alter the level of the 42 kDa beta actin monomer **[**24**]**.

In order to test whether these effects are unique to HUVEC or may also occur in other cell types, we downregulated MAGI1 in 293 T cells (Figure S4a) and monitored focal adhesion and actin stress fiber formation by immunostaining of paxillin and fibrillar actin, respectively. Consistent with the observation in HUVECs, downregulation of MAGI1 reduced the total number of paxillin-positive focal adhesions (Figure S4b) and decreased phalloidin-positive stress fibers (Figure S4c).

From these observations, we conclude that MAGI1 promotes the actomyosin-dependent formation or large (mature) focal adhesions and contributes to endothelial cell spreading.

### MAGI1 enhances endothelial cell adhesion to ECM proteins and promotes RhoA and Rac1 activation

**Figure 4.**
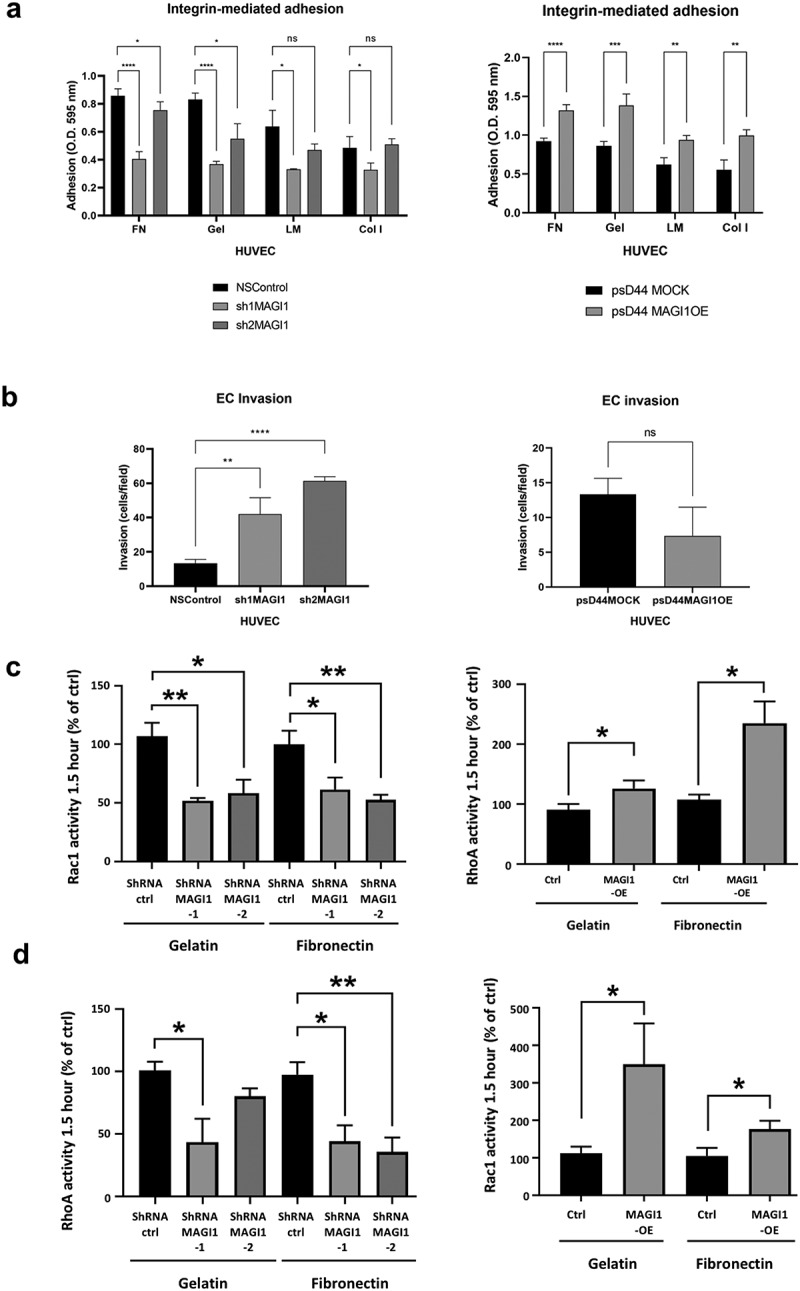
MAGI1 modulates endothelial cell adhesion, invasion and RhoA/Rac1 activity in vitro. (a). Integrin-mediated adhesion to ECM ligands fibronectin (FN), gelatin (Gel), laminin (LM), collagen I (Col I) of HUVEC with downregulated or overexpressed MAGI1 and their relative controls, as indicated. The data represent mean values ± S.D. (n = 3). (b). Invasion through Matrigel of HUVEC with downregulated or overexpressed MAGI1 and their relative controls, as indicated. The results represent the average number of cells per field ± S.D. (n = 3). (c-d). (c) RhoA and (d) Rac1 relative activity in HUVEC with silenced or overexpressed MAGI1 (and relative controls) 90 minutes after plating on both gelatin and fibronectin, as indicated (n = 3)

Sprouting angiogenesis requires endothelial cell migration, which in turn depends on the continuous formation and disassembly of focal adhesions **[**36,37**]**. As MAGI1 promoted maturation of stable focal adhesions, we hypothesized that MAGI1 could modulate adhesion to the ECM and by consequence cell migration. To test this hypothesis, we first monitored the effect of MAGI1 silencing and overexpression on integrin-dependent HUVEC adhesion to the ECM proteins fibronectin, collagen I, laminin and gelatin, four integrin ECM ligands relevant to angiogenesis **[**4**]**. MAGI1 overexpression promoted cell adhesion on all these ECM proteins, while MAGI1 silencing blunted it (Figure 4a). Next, we tested the effect of MAGI1 silencing and overexpression on HUVEC invasion through Matrigel, and observed that HUVEC with MAGI1 downregulation were less invasive, while HUVEC overexpressing MAGI1 were less invasive, compared to their respective controls (Figure 4b).

The small GPTases RhoA and Rac1 play a critical role in regulating focal adhesion maturation, actin remodeling, cell adhesion and migration **[**38**]**. To assess whether MAGI1 may modulate RhoA and Rac1 functions, we performed a GTPAse activity assay on HUVEC with silenced or overexpressed MAGI1 (and their relative controls), freshly plated on gelatin and fibronectin (HUVEC adhere to gelatin via αVβ3 integrins and to fibronectin with αVβ3 and α5β1 **[**39**]**). Ninety minutes after plating, HUVEC with silenced MAGI1 had lower levels of active Rac1 and RhoA compared to non-silenced HUVEC, on both gelatin and fibronectin substrates. Conversely, overexpression of MAGI1 enhanced both RhoA and Rac1 activities, particularly on gelatin (Figure 4c and 4d). These MAGI1 effects on RhoA and Rac1 activity were no longer significant after 24 hours of culture (not shown), consistent with an effect dependent on (synchronized) integrin mediated adhesion.

These results demonstrate that MAGI1 activity promotes integrin-mediated endothelial cell adhesion and RhoA and Rac1 activation, whil it inhibits endothelial cell invasion.

### MAGI1 overexpression reduces endothelial cell tubulogenesis in vitro and angiogenesis in vivo

**Figure 5.**
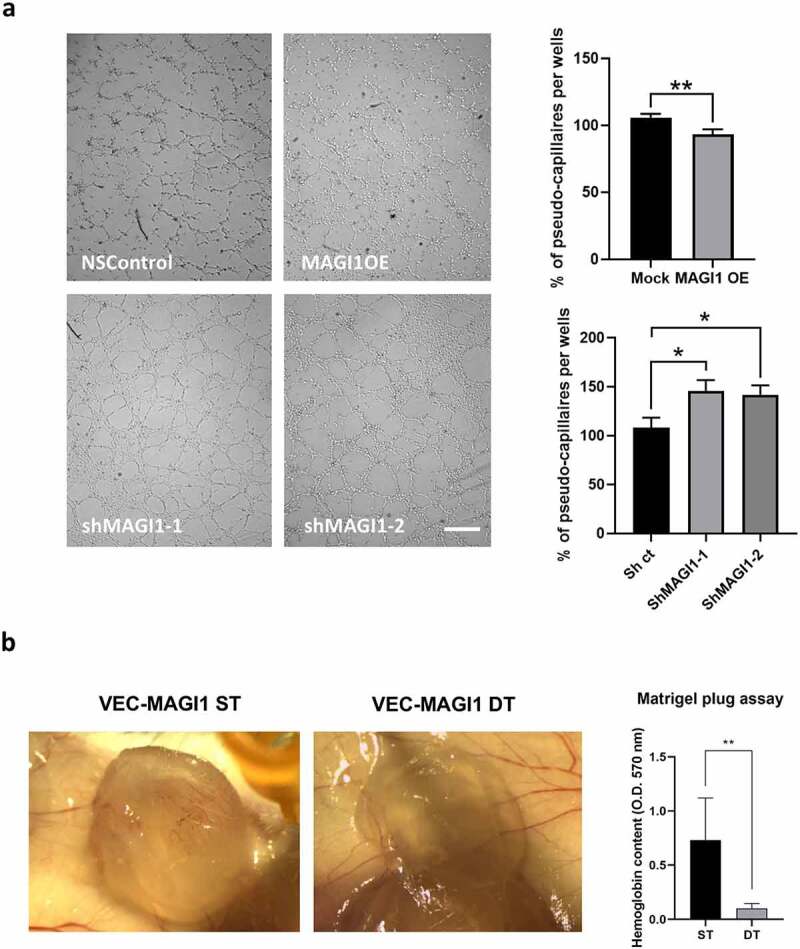
MAGI1 suppresses pseudocapillary formation *in vitro* and angiogenesis *in vivo*. (a). Representative images (left) of *in vitro* tubulogenesis assay of HUVEC with silenced (shMAG1-1 and shMAG1-2) or overexpressing (MAGI1OE) MAGI1 plated on polymerized Matrigel for 4 hours and quantification (right) of pseudocapillary formation. (n = 3). Scale bars correspond to 200 µm. (b). Representative images of Matrigel plugs (left) from single transgenic control (ST, tetOS::MAGI1) and double transgenic MAGI1OE (DT, VEC:tTA::tetOS:MAGI1) mouse line and quantification (right) of Matrigel plugs by measurement of hemoglobin content. * denotes angiogenic vessels in the Matrigel plug. The data represents mean values ± S.D. (n = 6). Statistical analyses were performed by unpaired t-test. ns = no statistically significant difference, * P < 0.05, *** P < 0.005, **** P < 0.001

Cell adhesion, invasion and Rho family GTPases activity are critical events for angiogenesis **[**37,40**]**. We therefore assessed whether MAGI1 modulation would impact angiogenesis. Firstly, we tested the effect of MAGI1 downregulation and overexpression in pseudocapillary network formation using an *in vitro* tubulogenesis assay **[**28**]**. MAGI1 downregulation promoted, while MAGI1 overexpression reduced pseudocapillary and endothelial network formation (Figure 5a). Secondly, we tested whether MAGI1 may regulate angiogenesis *in vivo* using the Matrigel plug assay **[**30**]**. To this end we used a double transgenic mouse line (DT, VEC:tTA::tetOS:MAGI1) that constitutively overexpress transgenic MAGI1 in endothelial cells in the absence of doxycycline, and its single transgenic control (ST, tetOS::MAGI1) that does not express the MAGI1 transgene **[**24**]**. Consistently, we observed that VEC-MAGI1 overexpressing DT mice have reduced angiogenesis compared to ST control mice, as revealed by measurements of hemoglobin content in the Matrigel plug **[**29**]** (Figure 5b).

Taken together, these results indicate that MAGI1 acts as a suppressor of endothelial pseudocapillary formation *in vitro* and sprouting angiogenesis *in vivo*.

### MAGI1 co-immunoprecipitates with paxillin and regulates focal adhesion turnover

**Figure 6.**
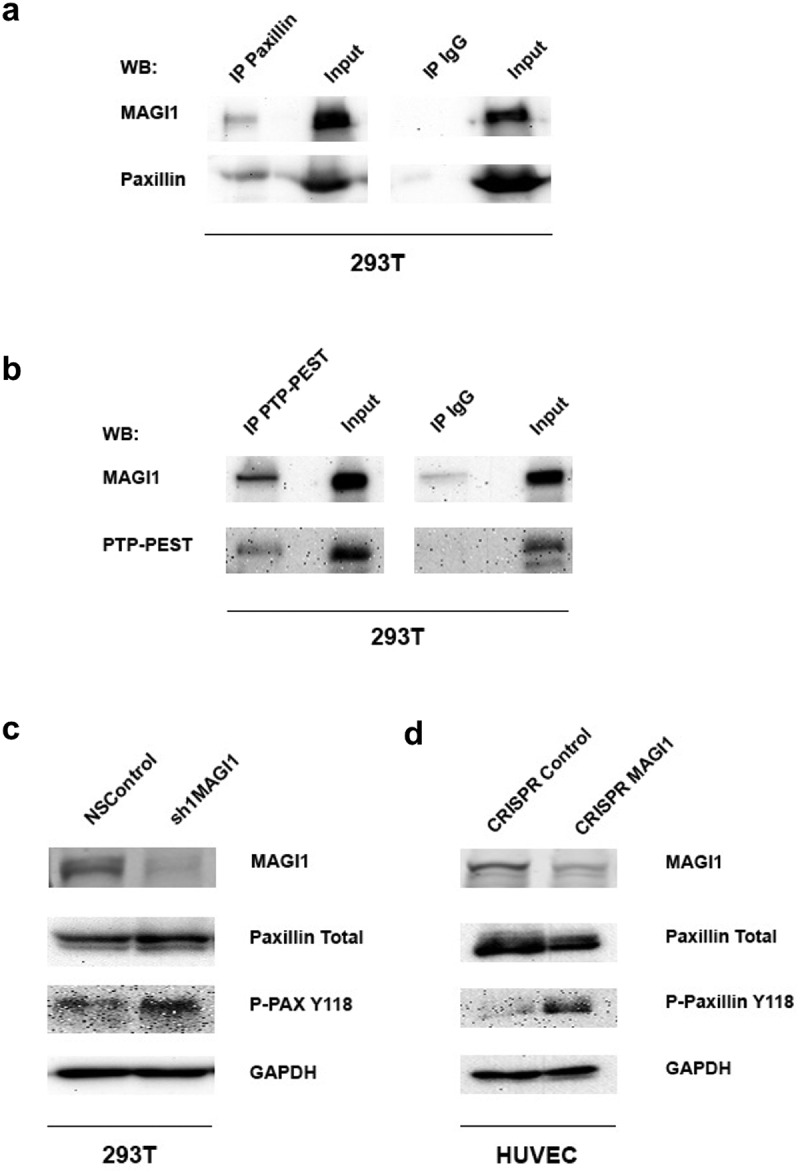
MAGI1 co-immunoprecipitates with paxillin and PTP-PEST and regulates phosphorylation paxillin Y118 residue. (a-b) Co-immunoprecipitation of MAGI1 with (a) paxillin and (b) PTP-PEST in 293 T cells. (c-d). Western blot of MAGI1, paxillin total, phospho–paxillin Y118 and GAPDH in (c) 293 T cells (ratio phospho paxillin shMAGI/NSControl [paxillin corrected]: 1,67: 1) and (d) HUVEC (ratio phospho paxillin CRISPR MAGI/CRISPR control [paxillin corrected]: 14.7: 1). GAPDH is shown as loading control

The above experiments demonstrated that MAGI1 regulates integrin–dependent endothelial cell spreading, adhesion, and migration/invasion, which require regulated focal adhesion dynamics, i.e. disassembly and turnover. Focal adhesion turnover is regulated by phosphorylation and dephosphorylation of tyrosine residues of proteins of the adhesome, most notably paxillin **[**36,41–43**]**. Phosphatase PTP-PEST is known to bind to and act on paxillin, thereby regulating cell spreading and motility **[**44,45**]**. We therefore tested whether MAGI1 associates with paxillin and PTP-PEST. Co-immunoprecipitation experiments indeed confirmed that MAGI1 interacts with paxillin (Figure 6a) and phosphatase PTP-PEST (Figure 6b) in 293 T cells indicating a possible functional connection.

Next, we looked for the phosphorylation state of paxillin and found that downregulation of MAGI1 in 293 T cells (Figure 6c) and in HUVEC (Figure 6d) increases phosphorylation of paxillin at Y118, consistent with increased focal adhesion turnover **[**41**]**.

These results indicate that downregulation of MAGI1 increases phosphorylation of paxillin at Y118 leading to higher focal adhesion turnover rates and unstable and dynamic focal adhesions.

## Discussion

Considering that MAGI1 functions as an adaptor protein at tight and adherens junctions in cells of epithelial and endothelial origin [15–19,23,24], and that it interacts the actin associated proteins such as α-4-actinin and synaptopodin where it links tight junctions to the cytoskeleton [18], we sought to investigate whether MAGI1 might be linking the cytoskeleton to other adhesive structures such as integrin-containing focal adhesions. Double immunostaining shows that MAGI1 colocalizes with phospho-paxillin, β3-integrin, talin 1 and tensin 3 at large focal adhesions but not at the small dot-like and dynamic lamellipodial focal complexes [10] nor at the centripetally located β1-contaning fibrillar adhesions [31,32]. We also show that MAGI1 is present with talin 1 and β3-integrin in immunoprecipitated protein complexes. These results suggest that MAGI1 is recruited to the adhesome at later steps of focal adhesion maturation. Consistently, experimental downregulation of MAGI1 in HUVEC leads to a reduction in the number of paxillin-positive focal adhesions, especially of the growing and large ones. MAGI1 overexpression facilitated focal adhesion maturation but the effect was statistically non-significant. This discrepancy could be due to the fact that MAGI1 expression in HUVEC is already high and further expression has only a mild effect further masked by intercellular variability. Alternatively, or concurrently, the expressed MAGI1b splice variant may not be the most effective of the three MAGI1 isoforms (i.e. MAGI1a, 1b, 1 c) [17] in regulating focal adhesion dynamics.

Focal adhesion maturation involves the recruitment of several cytoplasmic proteins such as zyxin, α-4-actinin or tensin 3 to the adhesome [11,41,46] which are critical for FA translocation from polymerized cortical actin in the lamellipodium to actin stress fibers at the lamellum [8,47]. Consistently, we observed that MAGI1 colocalizes with actin along stress fibers, but not with cortical actin, and with the actin associated protein α-4-actinin along actin stress fibers, and at the junctions between large focal adhesions and actin stress fibers (at the edges of lamellas). MAGI1 interaction with α-4-actinin has been already reported in podocytes, but in the context of tight junctions [18]. Here, by triple immunostaining, we demonstrate for the first time that MAGI1 colocalizes with α-4-actinin and paxillin at mature focal adhesions.

In line with the observation that MAGI1 is an actin-associated protein at actin stress fibers, HUVECs with downregulated MAGI1 displayed aberrant and tangled actin stress fibers and cells were unable to spread and remained smaller and more circular. These results suggest that by stabilizing focal adhesions and actin stress fibers, MAGI1 contributes to the generation of the tension necessary for focal adhesion maturation and the maintenance of a contractile and spread phenotype [8,48]. Consistent with a role in regulating actin dynamic, we observed that upon integrin engagement MAGI1 modulates the activity of RhoA and Rac1, two small GTPases that control cytoskeleton dynamics [38].

Mature focal adhesions play also a role during cell anchorage to the substrate. On the other hand, cell migration is sustained by the dynamic formation and disassembly of nascent adhesions [35]). For this reason, MAGI1 effects on focal adhesion dynamics could translate into functional changes in endothelial cells. Indeed, we observed that MAGI1 overexpression promotes HUVEC adhesion to different ECM ligands while MAGI1 downregulation decreased endothelial cell adhesion and pseudocapillary formation, while it increases invasion through Matrigel. Consistent with these *in vitro* observations, overexpression in endothelial cell *in vivo* in transgenic mice reduced angiogenesis in the Matrigel plug assay.

These observations raised the question about the mechanism by which MAGI1 regulates focal adhesion dynamics. As paxillin is a key regulator of focal adhesion dynamics [49] we tested whether MAGI1 regulates paxillin dynamics at focal adhesions. We found that MAGI1 co-immunoprecipitates with paxillin and the phosphatase PTP-PEST, one of the main regulators of paxillin de-phosphorylation at focal adhesions [44,45,48]. Interestingly, cells with MAGI1 downregulation have increased levels of phosphorylated paxillin at Tyr118. In dynamic adhesions, paxillin is phosphorylated at Tyr31 and Tyr118 by the FAK/Src complex leading to high turnover of focal complexes, translocation of focal adhesions and induction of lamellipodial protrusions which ultimately promote cell migration [36,50,51].

In conclusion, with this study we report a novel function for the scaffolding protein MAGI1 in endothelial cells: MAGI1 localizes at mature focal adhesions and actin stress fibers, and promotes focal adhesion maturation and reduces its turnover. At the cellular level, these MAGI1 effects translate into an increased EC adhesion to the ECM, reduced EC invasion *in vitro* and decreased pseudocapillary formation *in vitro* and angiogenesis *in vivo*.

## Supplementary Material

Supplemental Material

## References

[1] Bazzoni G, Dejana E. Endothelial cell-to-cell junctions: molecular organization and role in vascular homeostasis. Physiol Rev. 2004;84(3):869–901. 15269339 10.1152/physrev.00035.2003

[2] Wu MH. Endothelial focal adhesions and barrier function. J Physiol. 2005;569(2):359–366. 16195317 10.1113/jphysiol.2005.096537 PMC1464245

[3] Sun Z, Guo SS, Fassler R. Integrin-mediated mechanotransduction. J Cell Biol. 2016;215(4):445–456. 27872252 10.1083/jcb.201609037 PMC5119943

[4] Alday-Parejo B, Stupp R, Ruegg C. Are integrins still practicable targets for anti-cancer therapy? Cancers (Basel). 2019;11(7):978. 10.3390/cancers11070978 PMC6678560 31336983

[5] Calderwood DA. Integrin activation. J Cell Sci. 2004;117(5):657–666. 14754902 10.1242/jcs.01014

[6] Wolfenson H, Henis YI, Geiger B, et al. The heel and toe of the cell’s foot: a multifaceted approach for understanding the structure and dynamics of focal adhesions. Cell Motil Cytoskeleton. 2009;66(11):1017–1029. 19598236 10.1002/cm.20410 PMC2938044

[7] Petit V, Thiery JP. Focal adhesions: structure and dynamics. Biol Cell. 2000;92(7):477–494. 11229600 10.1016/s0248-4900(00)01101-1

[8] Parsons JT, Horwitz AR, Schwartz MA. Cell adhesion: integrating cytoskeletal dynamics and cellular tension. Nat Rev Mol Cell Biol. 2010;11(9):633–643. 20729930 10.1038/nrm2957 PMC2992881

[9] Sun Z, Lambacher A, Fassler R. Nascent adhesions: from fluctuations to a hierarchical organization. Curr Biol. 2014;24(17):R801–803. 25202871 10.1016/j.cub.2014.07.061

[10] Zimerman B, Volberg T, Geiger B. Early molecular events in the assembly of the focal adhesion-stress fiber complex during fibroblast spreading. Cell Motil Cytoskeleton. 2004;58(3):143–159. 15146534 10.1002/cm.20005

[11] Vicente-Manzanares M, Horwitz AR. Adhesion dynamics at a glance. J Cell Sci. 2011;124(23):3923–3927. 22194302 10.1242/jcs.095653 PMC3244977

[12] Romer LH, Birukov KG, Garcia JG. Focal adhesions: paradigm for a signaling nexus. Circ Res. 2006;98(5):606–616. 16543511 10.1161/01.RES.0000207408.31270.db

[13] Wolfenson H, Lavelin I, Geiger B. Dynamic regulation of the structure and functions of integrin adhesions. Dev Cell. 2013;24(5):447–458. 23484852 10.1016/j.devcel.2013.02.012 PMC3878073

[14] Dobrosotskaya I, Guy RK, James GL. MAGI-1, a membrane-associated guanylate kinase with a unique arrangement of protein-protein interaction domains. J Biol Chem. 1997;272(50):31589–31597. 9395497 10.1074/jbc.272.50.31589

[15] Ide N, Hata Y, Nishioka H, et al. Localization of membrane-associated guanylate kinase (MAGI)-1/BAI-associated protein (BAP) 1 at tight junctions of epithelial cells. Oncogene. 1999;18(54):7810–7815. 10618722 10.1038/sj.onc.1203153

[16] Hirabayashi S, Tajima M, Yao I, et al. JAM4, a junctional cell adhesion molecule interacting with a tight junction protein, MAGI-1. Mol Cell Biol. 2003;23:4267–4282. 12773569 10.1128/MCB.23.12.4267-4282.2003 PMC156145

[17] Laura RP, Ross S, Koeppen H, et al. MAGI-1: a widely expressed, alternatively spliced tight junction protein. Exp Cell Res. 2002;275(2):155–170. 11969287 10.1006/excr.2002.5475

[18] Patrie KM, Drescher AJ, Welihinda A, et al. Interaction of two actin-binding proteins, synaptopodin and alpha-actinin-4, with the tight junction protein MAGI-1. J Biol Chem. 2002;277:30183–30190. 12042308 10.1074/jbc.M203072200

[19] Chastre E, Abdessamad M, Kruglov A, et al. TRIP6, a novel molecular partner of the MAGI-1 scaffolding molecule, promotes invasiveness. Faseb J. 2009;23:916–928. 19017743 10.1096/fj.08-106344

[20] Dobrosotskaya IY, James GL. MAGI-1 interacts with β-Catenin and is associated with cell–cell adhesion structures. Biochem Biophys Res Commun. 2000;270(3):903–909. 10772923 10.1006/bbrc.2000.2471

[21] Zaric J, Joseph JM, Tercier S, et al. Identification of MAGI1 as a tumor-suppressor protein induced by cyclooxygenase-2 inhibitors in colorectal cancer cells. Oncogene. 2012;31:48–59. 21666716 10.1038/onc.2011.218

[22] Kotelevets L, Van Hengel J, Bruyneel E, et al. Implication of the MAGI-1b/PTEN signalosome in stabilization of adherens junctions and suppression of invasiveness. Faseb J. 2005;19(1):115–117. 15629897 10.1096/fj.04-1942fje

[23] Sakurai A, Fukuhara S, Yamagishi A, et al. MAGI-1 is required for Rap1 activation upon cell-cell contact and for enhancement of vascular endothelial cadherin-mediated cell adhesion. Mol Biol Cell. 2006;17(2):966–976. 16339077 10.1091/mbc.E05-07-0647 PMC1356604

[24] Ghimire K, Zaric J, Alday-Parejo B, et al. MAGI1 mediates eNOS activation and NO production in endothelial cells in response to fluid shear stress. Cells. 2019;8(5):388. 10.3390/cells8050388 PMC6562810 31035633

[25] Bieler G, Hasmim M, Monnier Y, et al. Distinctive role of integrin-mediated adhesion in TNF-induced PKB/Akt and NF-κB activation and endothelial cell survival. Oncogene. 2007;26(39):5722–5732. 17369858 10.1038/sj.onc.1210354

[26] Horzum U, Ozdil B, Pesen-Okvur D. Step-by-step quantitative analysis of focal adhesions. MethodsX. 2014;1:56–59. 26150935 10.1016/j.mex.2014.06.004 PMC4472847

[27] Kim DH, Wirtz D. Focal adhesion size uniquely predicts cell migration. Faseb J. 2013;27(4):1351–1361. 23254340 10.1096/fj.12-220160 PMC3606534

[28] Ponce ML. Tube formation: an in vitro matrigel angiogenesis assay. Methods Mol Biol. 2009;467:183–188. 19301671 10.1007/978-1-59745-241-0_10

[29] Imaizumi N, Monnier Y, Hegi M, et al. Radiotherapy suppresses angiogenesis in mice through TGF-βRI/ALK5-dependent inhibition of endothelial cell sprouting. PLoS One. 2010;5(6):e11084. 20552031 10.1371/journal.pone.0011084 PMC2884035

[30] Nowak-Sliwinska P, Alitalo K, Allen E, et al. Consensus guidelines for the use and interpretation of angiogenesis assays. Angiogenesis. 2018;21:425–532. 29766399 10.1007/s10456-018-9613-x PMC6237663

[31] Pankov R, Cukierman E, Katz BZ, et al. Integrin dynamics and matrix assembly: tensin-dependent translocation of alpha(5)beta(1) integrins promotes early fibronectin fibrillogenesis. J Cell Biol. 2000;148(5):1075–1090. 10704455 10.1083/jcb.148.5.1075 PMC2174533

[32] Clark K, Pankov R, Travis MA, et al. A specific alpha5beta1-integrin conformation promotes directional integrin translocation and fibronectin matrix formation. J Cell Sci. 2005;118(2):291–300. 15615773 10.1242/jcs.01623 PMC3329624

[33] Mogilner A, Keren K. The shape of motile cells. Curr Biol. 2009;19(17):R762–771. 19906578 10.1016/j.cub.2009.06.053 PMC2864320

[34] Blangy A. Tensins are versatile regulators of Rho GTPase signalling and cell adhesion. Biol Cell. 2017;109(3):115–126. 27748980 10.1111/boc.201600053

[35] Beningo KA, Dembo M, Kaverina I, et al. Nascent focal adhesions are responsible for the generation of strong propulsive forces in migrating fibroblasts. J Cell Biol. 2001;153:881–888. 11352946 10.1083/jcb.153.4.881 PMC2192381

[36] Webb DJ, Donais K, Whitmore LA, et al. FAK-Src signalling through paxillin, ERK and MLCK regulates adhesion disassembly. Nat Cell Biol. 2004;6(2):154–161. 14743221 10.1038/ncb1094

[37] Lamalice L, Le Boeuf F, Huot J. Endothelial cell migration during angiogenesis. Circ Res. 2007;100(6):782–794. 17395884 10.1161/01.RES.0000259593.07661.1e

[38] Schoenwaelder SM, Burridge K. Bidirectional signaling between the cytoskeleton and integrins. Curr Opin Cell Biol. 1999;11(2):274–286. 10209151 10.1016/s0955-0674(99)80037-4

[39] Dormond O, Foletti A, Paroz C, et al. NSAIDs inhibit αVβ3 integrin-mediated and Cdc42/Rac-dependent endothelial-cell spreading, migration and angiogenesis. Nat Med. 2001;7(9):1041–1047. 11533708 10.1038/nm0901-1041

[40] Barlow HR, Cleaver O. Building blood vessels—one Rho GTPase at a time. Cells. 2019;8(6):545. 10.3390/cells8060545 PMC6627795 31174284

[41] Zaidel-Bar R, Milo R, Kam Z, et al. A paxillin tyrosine phosphorylation switch regulates the assembly and form of cell-matrix adhesions. J Cell Sci. 2007;120(1):137–148. 17164291 10.1242/jcs.03314

[42] Burridge K, Sastry SK, Sallee JL. Regulation of cell adhesion by protein-tyrosine phosphatases. I. Cell-matrix adhesion. J Biol Chem. 2006;281(23):15593–15596. 16497668 10.1074/jbc.R500030200

[43] Deakin NO, Turner CE. Paxillin comes of age. J Cell Sci. 2008;121(15):2435–2444. 18650496 10.1242/jcs.018044 PMC2522309

[44] Shen Y, Schneider G, Cloutier JF, et al. Direct association of protein-tyrosine phosphatase PTP-PEST with paxillin. J Biol Chem. 1998;273(11):6474–6481. 9497381 10.1074/jbc.273.11.6474

[45] Jamieson JS, Tumbarello DA, Halle M, et al. Paxillin is essential for PTP-PEST-dependent regulation of cell spreading and motility: a role for paxillin kinase linker. J Cell Sci. 2005;118(24):5835–5847. 16317044 10.1242/jcs.02693

[46] Haynie DT. Molecular physiology of the tensin brotherhood of integrin adaptor proteins. Proteins: Structure, Function, and Bioinformatics. 2014;82(7):1113–1127. 10.1002/prot.24560 24634006

[47] Choi CK, Vicente-Manzanares M, Zareno J, et al. Actin and α-actinin orchestrate the assembly and maturation of nascent adhesions in a myosin II motor-independent manner. Nat Cell Biol. 2008;10(9):1039–1050. 19160484 10.1038/ncb1763 PMC2827253

[48] Burridge K, Wittchen ES. The tension mounts: stress fibers as force-generating mechanotransducers. J Cell Biol. 2013;200(1):9–19. 23295347 10.1083/jcb.201210090 PMC3542796

[49] Qin R, Schmid H, Munzberg C, et al. Phosphorylation and turnover of paxillin in focal contacts is controlled by force and defines the dynamic state of the adhesion site. Cytoskeleton (Hoboken). 2015;72(2):101–112. 25620625 10.1002/cm.21209

[50] Zaidel-Bar R, Ballestrem C, Kam Z, et al. Early molecular events in the assembly of matrix adhesions at the leading edge of migrating cells. J Cell Sci. 2003;116(22):4605–4613. 14576354 10.1242/jcs.00792

[51] Lopez-Colome AM, Lee-Rivera I, Benavides-Hidalgo R, et al. Paxillin: a crossroad in pathological cell migration. J Hematol Oncol. 2017;10(1):50. 28214467 10.1186/s13045-017-0418-y PMC5316197

