# Supplementary material for: MAGI1 localizes to mature focal adhesion and modulates endothelial cell adhesion, migration and angiogenesis
Source: Cell Adh Migr. 2021 May 6;15(1):126–39. doi: 10.1080/19336918.2021.1911472 (PMC8115569; doi:10.1080/19336918.2021.1911472)
Supplement: Supplemental Material [file KCAM_A_1911472_SM8534.zip › KCAM-2019-0044_Suppl MM_REV.pdf]

Supplementary materials and methods

Western Blotting

Proteins were extracted in cooled RIPA buffer containing protease inhibitor mix (Sigma-Aldrich) and the following phosphatase inhibitors: phenylmethylsulfonylfluoride (PMSF), sodium orthovanadate (Na3VO4) and glycerophosphate disodium salt hydrate (BGP). Protein lysates were then sonicated and centrifuged at 13000 rpm for 10 minutes at 4°C and supernatants were collected. Protein concentration was calculated by using a BSA standard curved and Bradford assay (Bio-Rad). Proteins were resolved under reducing conditions by SDS-PAGE (sodium dodecyl sulfate polyacrylamide gel electrophoresis) in 9% polyacrylamide gels. Subsequently, proteins were transferred to PVDF Immobilon-FL membranes (Merck Millipore) typically for 60-90 minutes at 100V and 250 mA. Membranes were then blocked with 5% BSA for 1 hour at room temperature and incubated at 4°C overnight with the corresponding primary antibodies. Next, membranes were washed and then incubated for 1 hour at room temperature with HRP-coupled secondary antibodies (1/2000; DAKO). Protein detection was performed with short incubation with the ECL reagent Luminata Classico Western HRP substrate (Merck Millipore).

Band intensity of proteins was quantified by using ImageJ software (National Institutes of Health, Bethesda, MD, USA), and the relative expression of protein was normalized to the expression of GAPDH (as loading control). Band quantification for phospho paxillin and total paxillin were performed with Image J and ratios phospho paxillin sh1 MAGI/NS Control and CRISPR MAGI/CRISPR Control, corrected for paxillin, were calculated. Rations are given in the figure legends.

Plasmids, Lentivirus Production and Transduction

Human MAGI1b cDNA was kindly provided by Dr Y. Hata (Dept. Medical Biochemistry, Graduate School of Medicine, Tokyo Medical and Dental University. Tokyo, Japan) and sub-cloned into pRRLSIN.cPPT.PGK/GFP.WPRE lentiviral vector under the control of the phospho glycerate kinase (PGK) promoter (= pSD44 plasmid). pLentiMOCK (pLenti-III-Blank) and pLentiMAGI1OE (pLenti-GIII-CMV-Cterm-HA) were purchased from Applied Biomedical Materials Inc. Both psD44MAGI1 and pLentiMAGI1 constructs contain cDNA of MAGI1b isoform and mammalian puromycin resistance. Additionally, pLentiMAGI1 construct contain an HA-tag.

For gene silencing experiments, the pCMV-GIN-ZEO lentiviral shRNAmir expressing system was purchased from Open Biosystems (Huntsville, AL) and two different clones were used: sh1MAGI1 (ID: V2LHS_36236) and sh2MAGI1 (ID: V2LHS_36239). The shRNAmir sequences were cloned into the pSHAG-MAGIC2 (pSM2c) retroviral vector. After, the two shRNAmir sequences, or a non-silencing control shRNAmir sequence that contains no homology to known mammalian genes, were subcloned

into the pCMV-GIN-ZEO lentiviral system containing the neomycin/geneticin resistance gene and a GFP-tag.

Lentivirus were produced in HEK293T cells by calcium phosphate precipitation. Briefly, two hours prior to transfection, $11x10^6$ HEK293T cells/15 cm dish were incubated with DMEM + 10%FCS supplemented with 25 µM chloroquine (Sigma-Aldrich). Lentiviruses were produced by co-transfection of HEK293T cells with the vectors of interest together with the pCMV-dR8.74 and the pMD2G (VSVG). The day after, media were replaced with fresh media containing 3 mM caffeine (Sigma-Aldrich). Next day, the supernatants containing the viral particles were collected, ultracentrifuged for 2 hours at 20000 rpm for lentivirus particles concentration and used to infect cells immediately, or aliquoted and stored at -80 °C until use.

Cell cultures were transduced by over-night incubation at 37 °C in virus-containing media in the presence of 10 µg/mL polybrene (Sigma-Aldrich). Selection was started 48 h after transduction using 1 µg/mL Puromycin (Sigma-Aldrich) for pSD44-MAGI1-cDNA construct or 250 µg/mL G418 (GIBCO, Life Technologies) for pCMV-GIN-ZEO constructs.

Generation of transgenic mice

The human MAGI1 cDNA (NM_004742.2, 3770bp) was inserted into the pTetOS responder construct to generate transgenic animals. Driver and responder transgenic animals were bred to generate bigenic mice. Offspring was genotyped to generate wild type, single (ST, tetOS:MAGI1 and VEC:tTA) and double transgenics (DT, VEC:tTA:: tetOS:MAGI1) mice. In the absence of doxycycline, mice constitutively overexpress transgenic MAGI1 in endothelial cells while in the presence of doxycycline transgenic expression of MAGI1 is silenced. Doxycycline treatment involved the addition of 100 µg/mL of doxycycline (cat. no. D9891, Sigma-Aldrich)/5% sucrose in the drinking water and was changed at least twice per week. Animals were euthanized by CO2 inhalation followed by neck dislocation. Animal experiments were approved by the Cantonal Office in Fribourg (2014_26_FR) and performed according to Swiss regulations and to the guidelines from Directive 2010/63/EU of the European Parliament on the protection of animals used for scientific purposes. We used both male and female mice between 6 and 10 weeks of age. The following primers were used for genotyping the mice: VEC_forward: 5′GACGCCTTAGCCATTGAGAT 3′, VEC_reverse: 5′CAGTAG TAG GTGTTTCCCTTTCTT 3′, MAGI1_forward: 5′ TCATTCCTGGGCATGAGTCCT 3′, MAGI1_reverse: 5`GCCAGGGAAGGAAGGATTGT3′.
