# Supplementary material for: MAGI1 localizes to mature focal adhesion and modulates endothelial cell adhesion, migration and angiogenesis
Source: Cell Adh Migr. 2021 May 6;15(1):126–39. doi: 10.1080/19336918.2021.1911472 (PMC8115569; doi:10.1080/19336918.2021.1911472)
Supplement: Supplemental Material [file KCAM_A_1911472_SM8534.zip › KCAM-2019-0044__Suppl_figure_and_legends_CLEAN.pdf]

Supplementary figures and legends

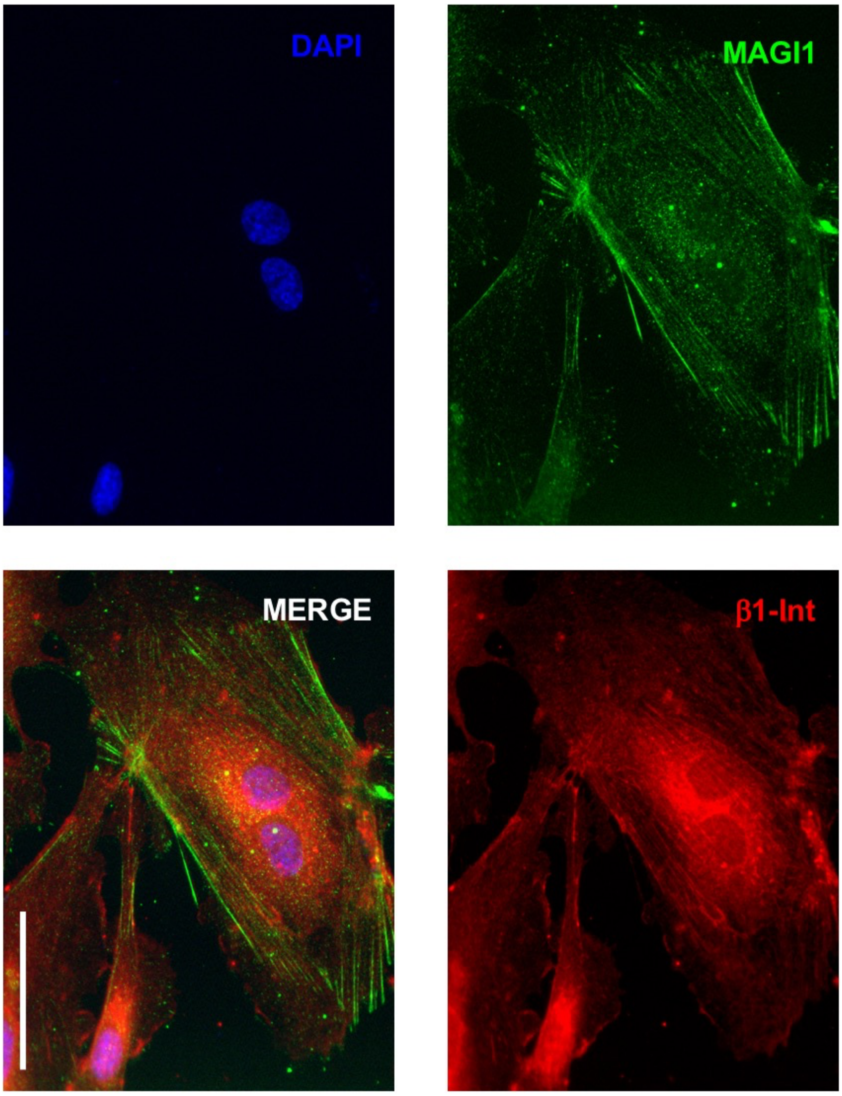

Figure S1. MAGI1 does not colocalize at fibrillar adhesions with β1-integrin. Representative fluorescence microscope images of immunofluorescence staining of MAGI1 (green), β1-integrin (red), DAPI (blue), and merged images in HUVEC (63x objective). Bar = 50 μm.

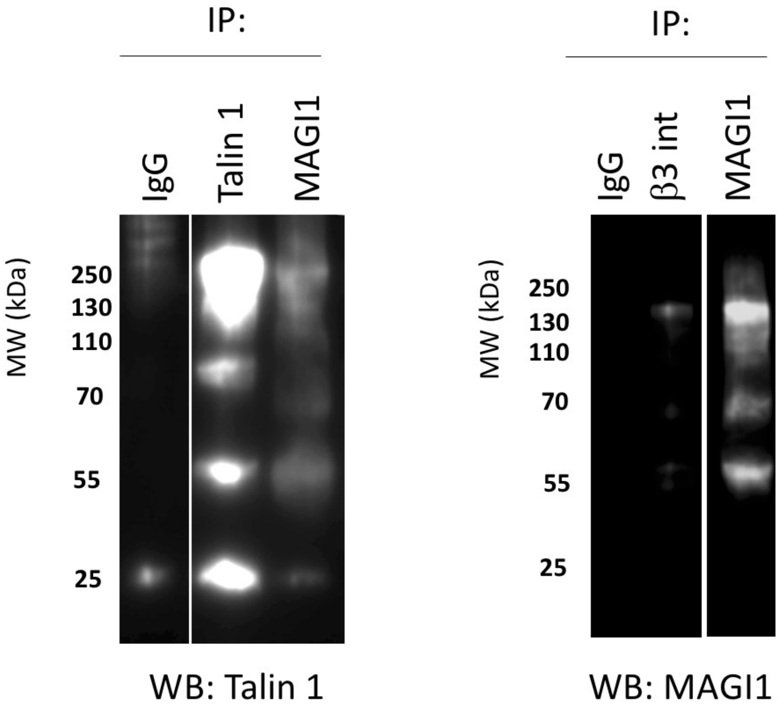

Figure S2. MAGI1 co-precipitates with β3-integrin and talin 1. Cell lysate obtained from adherent HUVECs was subjected to immunprecipitation with anti-talin 1, anti- MAGI1, anti-β3-integrin and an IgG control antibody, and the immunoprecipitated material was probed for talin 1 and MAGI1 by western blotting, as indicated. Bands at around 55 and 25 kDa correspond to H an L chains of primary antibodies. Numbers on the left indicate MW (kDa).

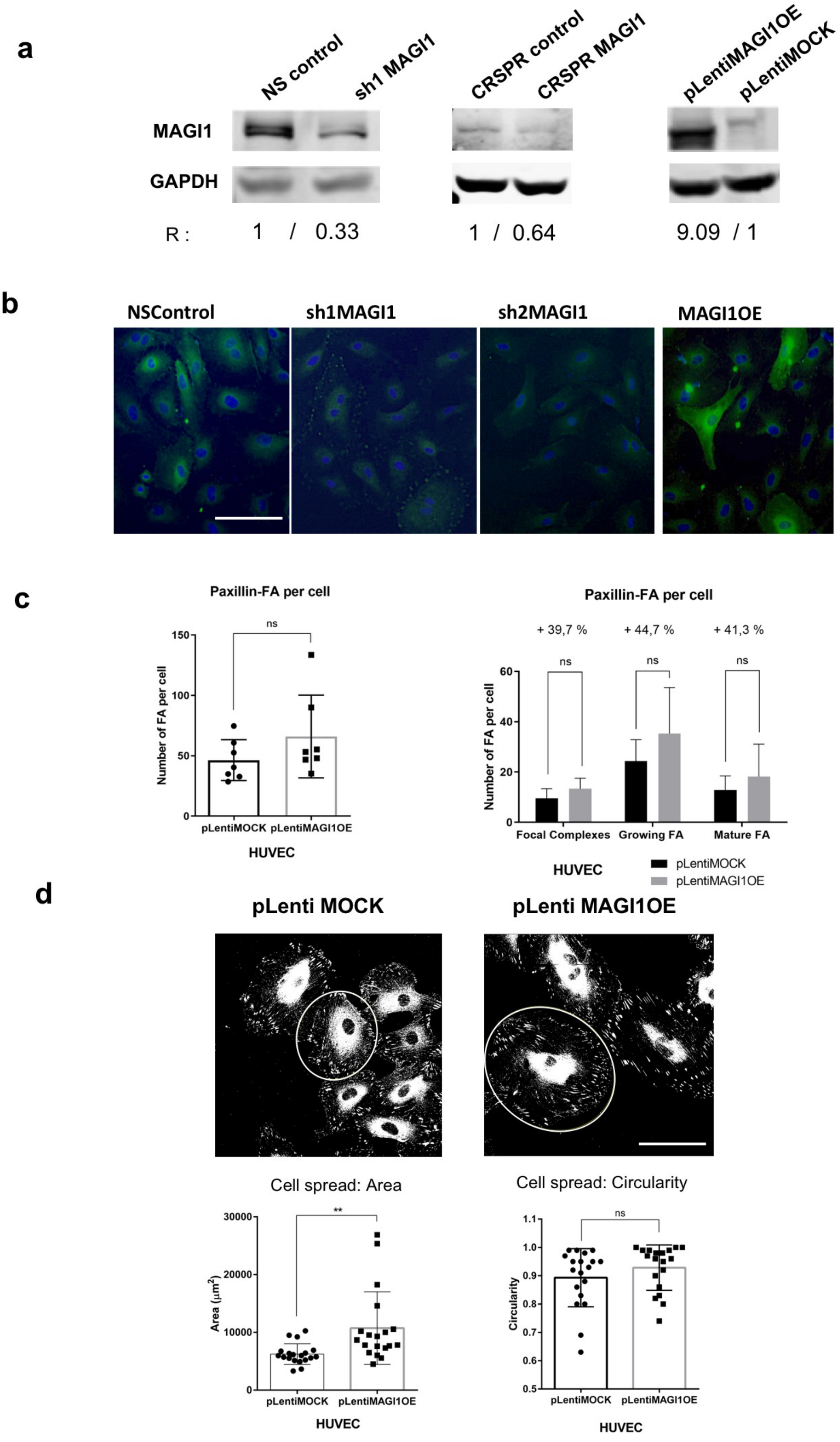

Figure S3. MAGI1 overexpression in HUVEC reduces focal adhesion formation and cell spread (a). Western blot of MAGI1 in HUVEC for the validation of MAGI1 downregulation (sh1MAGI1 and CRISPR MAGI1) and overexpression (pLentiMAGI1OE). GAPDH detection is used as loading control. (b). MAGI1 staining of control HUVEC (NSControl), HUVEC with MAGI1 downregulation (sh1MAGI1, sh2MAGI1) and HUVEC with MAGI1 overexpression (MAGI1OE) (n=2). Bar = 50 µm. (c). Quantification of total number of paxillin-positive focal adhesions (FA) per cell in HUVEC. (d). Quantification of number of paxillin-stained focal

adhesions (FA) per cell by group size in HUVEC. Nascent adhesions or focal complexes (FXs) (< 1 μm2), growing focal adhesions (FAs) (1 – 3 μm2) and mature FAs (> 3 μm2). The data represents mean values ± S.D. (n = 7). Bar = 50 μm (e). Representative images and quantification of cell spread area and circularity in paxillin-positive images of HUVEC (63x objective). The data represents mean values ± S.D. (n = 19). Statistical analyses were performed by unpaired t-test. ns = no statistical difference, * P < 0.05, *** P < 0.005, **** P < 0.001.

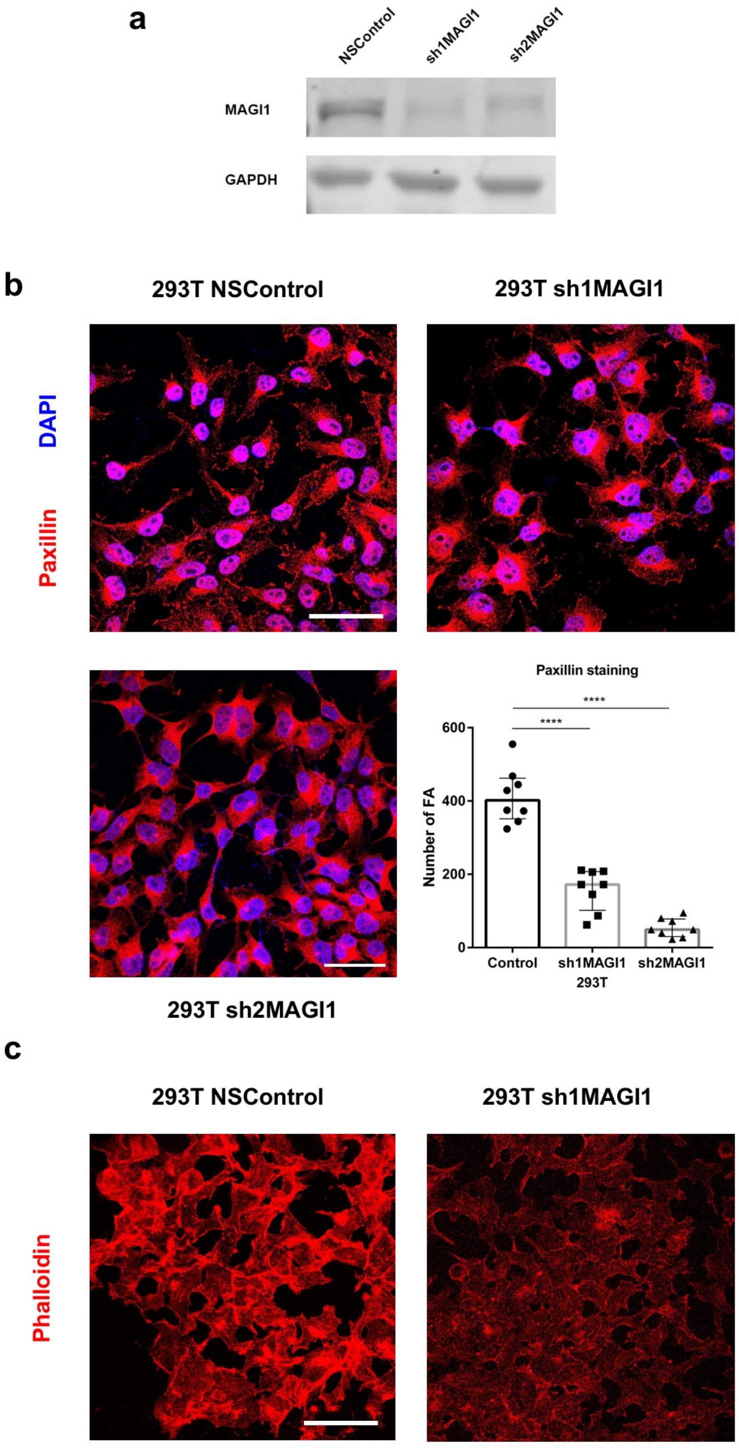

Figure S4. MAGI1 downregulation in 293T cells decreases the number of focal adhesions and decreases actin stress fiber formation. (a). Western blot of MAGI1 and GAPDH in 293T cells for the validation of MAGI1 downregulation (sh1MAGI1 and sh2MAGI1). GAPDH is used as loading control. (b). Representative confocal images of immunofluorescence staining of paxillin (red) and DAPI (blue) and quantification of total number of paxillin-positive focal adhesions (FA) per cell in 293T cells (63x objective). The data represents mean values ± S.D. (n = 8). Statistical analyses were performed by unpaired t-test. ns = no statistical difference, * P < 0.05, *** P < 0.005, **** P < 0.001. (c). Representative confocal images of immunofluorescence staining of Phalloidin (red) in 293T cells (63x objective). Bar = 50 µm.
